# Romanian Wild-Growing *Chelidonium majus*—An Emerging Approach to a Potential Antimicrobial Engineering Carrier System Based on AuNPs: In Vitro Investigation and Evaluation

**DOI:** 10.3390/plants13050734

**Published:** 2024-03-05

**Authors:** Adina-Elena Segneanu, Gabriela Vlase, Titus Vlase, Maria-Viorica Ciocalteu, Cornelia Bejenaru, Gabriela Buema, Ludovic Everard Bejenaru, Eugen Radu Boia, Andrei Dumitru, Simina Boia

**Affiliations:** 1 Institute for Advanced Environmental Research-West, University of Timisoara (ICAM-WUT), Oituz nr. 4, 300223 Timisoara, Romania; gabriela.vlase@e-uvt.ro (G.V.); titus.vlase@e-uvt.ro (T.V.); 2 Research Center for Thermal Analysis for Environmental Problems, West University of Timisoara, Pestalozzi St. 16, 300115 Timisoara, Romania; 3 Faculty of Pharmacy, University of Medicine and Pharmacy Craiova, St. Petru Rareș 2, 200349 Craiova, Romania; maria.ciocilteu@umfcv.ro (M.-V.C.); cornelia.bejenaru@umfcv.ro (C.B.); ludovic.bejenaru@umfcv.ro (L.E.B.); 4 National Institute of Research and Development for Technical Physics, 47 Mangeron Blvd., 700050 Iasi, Romania; gbuema@phys-iasi.ro; 5 Department of Ear, Nose, and Throat, Faculty of Medicine, “Victor Babeș” University of Medicine and Pharmacy Timisoara, 2 Eftimie Murgu Sq., 300041 Timisoara, Romania; 6 Faculty of Sciences, Physical Education and Informatics—Department of Medical Assistance and Physiotherapy, National University for Science and Technology Politehnica Bucharest, University Center of Pitesti, St. Targu din Vale 1, 110040 Pitesti, Romania; andrei.dumitru@upit.ro; 7 Department of Periodontology, Faculty of Dental Medicine, Anton Sculean Research Center for Periodontal and Peri-Implant Diseases, “Victor Babeș” University of Medicine and Pharmacy Timisoara, 2 Eftimie Murgu Sq., 300041 Timisoara, Romania; simina.boia@umft.ro

**Keywords:** great celandine, AuNPs, carrier system, secondary metabolites, antioxidant activity, dissolution profile

## Abstract

Novel nanotechnology based on herbal products aspires to be a high-performing therapeutic platform. This study reports the development of an original engineering carrier system that jointly combines the pharmacological action of *Chelidonium majus* and AuNPs, with unique properties that ensure that the limitations imposed by low stability, toxicity, absorption, and targeted and prolonged release can be overcome. The metabolite profile of Romanian wild-grown *Chelidonium majus* contains a total of seventy-four phytochemicals belonging to eight secondary metabolite categories, including alkaloids, amino acids, phenolic acids, flavonoids, carotenoids, fatty acids, sterols, and miscellaneous others. In this study, various techniques (XRD, FTIR, SEM, DLS, and TG/DTG) were employed to investigate his new carrier system’s morpho-structural and thermal properties. In vitro assays were conducted to evaluate the antioxidant potential and release profile. The results indicate 99.9% and 94.4% dissolution at different pH values for the CG-AuNPs carrier system and 93.5% and 85.26% for greater celandine at pH 4 and pH 7, respectively. Additionally, three in vitro antioxidant assays indicated an increase in antioxidant potential (flavonoid content 3.8%; FRAP assay 24.6%; and DPPH 24.4%) of the CG-AuNPs carrier system compared to the herb sample. The collective results reflect the system’s promising perspective as a new efficient antimicrobial and anti-inflammatory candidate with versatile applications, ranging from target delivery systems, oral inflammation (periodontitis), and anti-age cosmetics to extending the shelf lives of products in the food industry.

## 1. Introduction

*Chelidonium majus* (Papaveraceae family) is the sole representative of the *Chelidonium* genus in the Romanian flora and, respectively, in that of Europe [1]. Its common names include greater celandine, swallow-wort, rostopască, negelarită in Romanian, and bai-qu-cai in Chinese. Greater celandine (GC) has been recognized as a plant with both medicinal and toxic properties since ancient times, with mentions dating back to ancient Europe and in Chinese traditional medicine [2]. From Dioscorides, Pliny the Elder, and Galenus until the XIV century, greater celandine was recommended for ocular ailments. In medieval Europe, the plant was used also for treating ulcers, cutaneous eczema, jaundice, and colic. Paracelsus mentioned the benefits of this plant for treating hepato-biliary conditions. Currently, greater celandine is appreciated in traditional European medicine, especially in the central and eastern regions, being recognized for its exceptional therapeutic properties, especially in dermatological conditions (e.g., eczema, verrucae, circumscribed cutaneous carcinomas), hepato-biliary (anti-jaundice) conditions, chole cytopathies, biliary lithiasis, gastrointestinal spasms, eye infections, and inflammation [3,4,5,6]. Modern research has reported the presence of a large variety of biomolecules (alkaloids, flavonoids, carotenoids, lectins, phenolic acids, volatile oils, and others) and, thus, remarkable biological activity (antibacterial, antimicrobial, antifungal, antiviral, anti-inflammatory, antitumoral, anti-spasmodic, hepato-protective, analgesic, and immunomodulatory) [7,8,9,10,11,12,13,14,15].

Cutting-edge nanotechnology-based phytochemical carriers have emerged as promising candidates with highly improved in vivo activity due to the overcoming of the drawbacks (low bioavailability, chemical, and thermally stability, and selectivity) of conventional herbal formulations [16]. Engineering phytochemical carriers are the most successful approaches, with highly improved in vivo activity. These carriers effectively overcome all the challenges posed by conventional herbal formulations, including low bioavailability, selectivity, and chemical and thermal stability [17].

Of all the metallic nanoparticles, gold nanoparticles are particularly well-suited for various biomedical applications thanks to their unique properties, such as versatile tailored surfaces, excellent stability, easy cellular uptake, and minimal toxicity. As a result, current research addresses the design of novel drug delivery systems that can mitigate drug resistance in cancer therapy, bacterial resistance antibiotics, etc. [18,19,20,21,22,23,24,25,26,27].

On the other hand, it is noteworthy that, despite the outstanding therapeutic activity of *Chelidonium majus*, its overdose due to self-medication with various market herbal supplements can induce severe outcomes on liver physiological function. Therefore, avoiding this liability requires advanced herbal formulations and safety and control of dosage, leading to increased efficacity [28,29].

Accordingly, in this study, our approach to plant-derived natural products moves to a different level, using the renowned medicinal plant *Chelidonium majus* and AuNPs to achieve an innovative engineering carrier system with unique pharmacological activity.

The chemical, morpho-structural, and thermal properties; antioxidant potential; and in vitro release profile were studied systematically.

## 2. Results and Discussion

Numerous research studies have been conducted on the chemical composition and pharmacological activity of *Chelidonium majus*. Most of these studies have focused on specific phytoconstituent categories found in certain parts of the plant [3,4,5,6,7,8,10,30,31,32].

However, the plant’s origin has an essential role in increasing the development of plants, as well as in triggering different defense mechanisms against various biotic factors in their environments. Among the most dominant defense systems of plants against environmental stress factors is a plant’s ability to produce varied secondary metabolites and signaling molecules. Accordingly, discrepancies occur in the metabolic profiles of particular plants of different origins [17,33]. Furthermore, extraction parameters, such as solvent polarity, temperature, and pH, have a decisive impact on the phytochemical composition [34,35,36].

To this end, establishing a correlation between the biomolecules found in a plant and its therapeutic activity is an arduous task.

Moreover, few studies have been performed, and only on the alkaloid or phenolic contents of the Romanian *Chelidonium majus* wild plant [37,38]. Therefore, this study investigates the low metabolic profile of greater celandine using gas-chromatography coupled with mass spectroscopy (GC-MS) and electrospray ionization–quadrupole time-of-flight mass spectrometry (ESI-QTOF-MS) analysis. The phytochemicals were identified on the retention indices, in the Mass Spectral Library 2.0 database, and in the literature.

The biomolecules separated via GC-MS are presented in Figure S1 and Table 1.

Table 1 shows the main phytoconstituents identified via GC-MS analysis from the greater celandine sample.

The GC-MS analysis displays thirteen compounds, accounting for about 87% of the total peak area in the greater celandine sample (Figure S1).

### 2.1. Mass Spectrometry Analysis of Chelidonium majus Sample

The MS spectra (Figure S2) indicate the presence of numerous molecules, some of which were detected and assigned to different chemical classes (alkaloids, amino acids, phenolic acids, flavonoids, carotenoids, organic acids, fatty acids, sterols, and others) that corroborate the literature results [6,7,11,15,31,33,38,40,42,43].

The phytoconstituents identified via ESI–QTOF–MS analysis are presented in Table 2.

### 2.2. Screening and Classification of the Differential Phytoconstituents

A total of seventy-four biomolecules were identified and assigned to several categories of secondary metabolites: alkaloids (28.38%), amino acids (about 15%), phenolic acids (12.16%), flavonoids (6.75%), carotenoids (6.75%), fatty acids (5.4%), phytosterols (about 4%), terpenoids (1.75%), and miscellaneous others.

**Figure 1:**
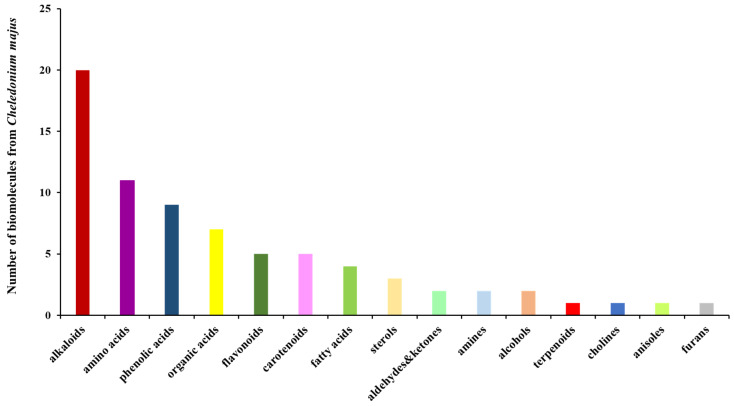
Biomolecule classification bar chart for *Chelidonium majus*.

Figure 1 shows the phytochemical classification chart from the *Chelidonium majus* sample based on the data analysis reported in Table 2.

According to Figure 1, alkaloids, the largest category of phytoconstituents, exhibited outstanding therapeutic activities: sedative, analgesic, antitumoral, antimicrobial, antifungal, anti-inflammatory, antidiabetic, antiemetic, antioxidant, neuroprotective, etc. [44,45].

In the greater celandine sample, eleven amino acids were identified, of which the largest percent (72.7%) was represented by non-essential amino acids (glycine, alanine, serine, proline, asparagine, aspartic acid, glutamic acid, and tyrosine). Essential amino acids (isoleucine, valine, and threonine) were present in a minor proportion (27.3%). Various research has reported the exceptional pharmacological activities of these phytochemicals (anti-inflammatory, neuroprotective, antiproliferative, cytotoxic, and immunomodulating activities [46,47,48,49,50,51,52,53]).

Phenolic acids made up about 12% of the biomolecules from the greater celandine sample, being involved in antioxidant, antimicrobial, cardioprotective, anti-inflammatory, neuroprotective, antitumor, and antidiabetic mechanisms [54].

Flavonoids (hyperoside, luteolin, quercetol, isorhamnetin, quercetin) are another secondary metabolite class with significant beneficial effects on human health (antioxidant, antimicrobial, antiviral, anti-inflammatory, antitumoral, antidiabetic, cardioprotective, hepatoprotective, and neuroprotective). In addition, luteolin is involved in the management of pain caused by anti-inflammatory disorders [55,56,57,58,59,60].

Recent research has shown that *carotenoids* exhibit antioxidant, anti-inflammatory, neuroprotective, cardioprotective, skin and eye protection, anti-obesity, antitumoral, and antimutagen activities [61,62].

*Fatty acids* represented 5.4% of the total phytoconstituents from the greater celandine sample. These secondary metabolites act as anti-inflammatory, antioxidant, cardioprotective, and neuroprotective agents [63,64].

*Phytosterols* represented 4.05% of the total phytochemicals from the greater celandine sample. These are involved in antioxidant, anti-inflammatory, immunomodulatory, antiatherosclerotic, neuroprotective, and antitumoral mechanisms [65].

The *terpenoid* limonene displays antitumoral, antimicrobial, antifungal, antidiabetic, anti-inflammatory, antiallergenic, antitumoral (breast tumor), and neuroprotective activities [66,67].

### 2.3. Phyto-Nanocarrier System

Advanced nanotechnology is the key to overcoming the limitations of biomedical applications of medicinal plant preparations with high therapeutic activity. The reduced stability, permeability, and bioavailability of some specific secondary metabolites in biological environments pose significant challenges [16,17,32]. However, the engineering nanocarriers based on metallic nanoparticles offer a highly effective solution by improving biocompatibility, reducing harmful side effects, and exhibiting higher therapeutic efficiency through the synergistic effect of both components, namely, drugs and metallic nanoparticles. Moreover, these tailored nanocarriers enhance stability, permeability, targeting control, and release. Therefore, the engineering nanocarriers used in biomedical applications represent a significant advancement and are poised to have promising potential in personalized therapeutic strategies [16,68]. Accordingly, a novel phyto-carrier based on preparation of AuNPs will synergically merge the biological activities of the *Chelidonium majus* biomolecules and metallic nanoparticles, which will achieve a higher therapeutic yield.

### 2.4. FT-IR and Raman Spectroscopy

Fourier transform infrared (FTIR) spectroscopy is a widely analytical technique used to obtain findings regarding molecular structure and chemical composition from complex matrices.

**Figure 2:**
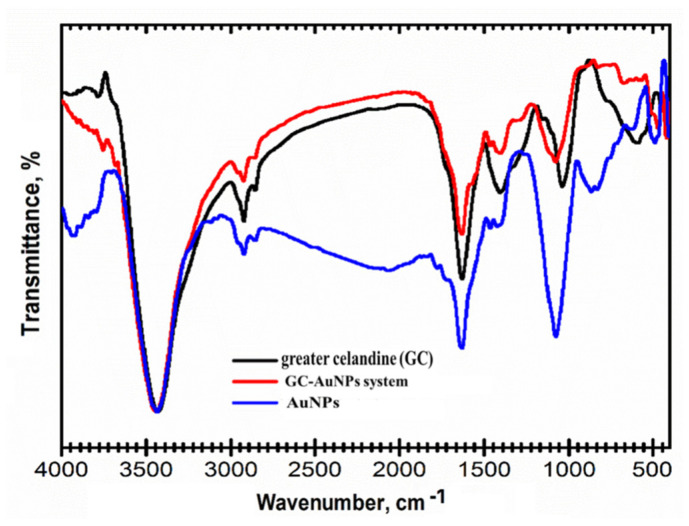
FT-IR overlap spectra of the carrier system of greater celandine, AuNPs, and GC-AuNPs.

Hence, the preparation of the GC-AuNPs carrier system was studied using FT-IR spectroscopy to emphasize the bonding mechanism between herbs and metallic nanoparticles. FT-IR spectra are presented in Figure 2.

The functional groups assigned to *Chelidonium majus* phytochemicals are shown in Table 3.

The FTIR spectra of the GC-AuNPs carrier system display the characteristic absorption bands of *Chelidonium majus* (3430 cm^−1^ (-OH group), 3293 cm^−1^ (O-H stretching carboxylic acid), 1709 cm^−1^ (C=O stretching vibration), 1609 cm^−1^ (C=C of carotenoids), 1601 cm^−1^ (C=C and C=N stretching vibrations of alkaloids), 1240 cm^−1^ (C-N of amine), 1032 cm^−1^ (NH stretching of amines), and 872 cm^−1^ (C-H bending vibration of aromatic rings)), as well as the AuNPs coated with trisodium citrate (2915 cm^−1^ (OH stretching vibration); 2848 cm^−1^ (corresponding to CH- asymmetric and symmetric stretching vibrations); 1596 cm^−1^ (COO- stretching vibration); and 1392 cm^−1^ (assigned to C–H bending)), thus pointing to the successful preparation of the CG-AuNPs carrier system [18,20,69,77].

Nonetheless, the changes in the intensity of the absorption bands and the shift toward higher wavenumbers in the corresponding regions (O-H, N-H, and C-H) are noticeable and indicate their involvement in the preparation of the GC-AuNPs carrier system [20,78,79].

### 2.5. Raman Spectroscopy

Raman spectroscopy is an important technique that is often used to study the vibrational modes of both molecules and hybrid nanomaterials.

**Figure 3:**
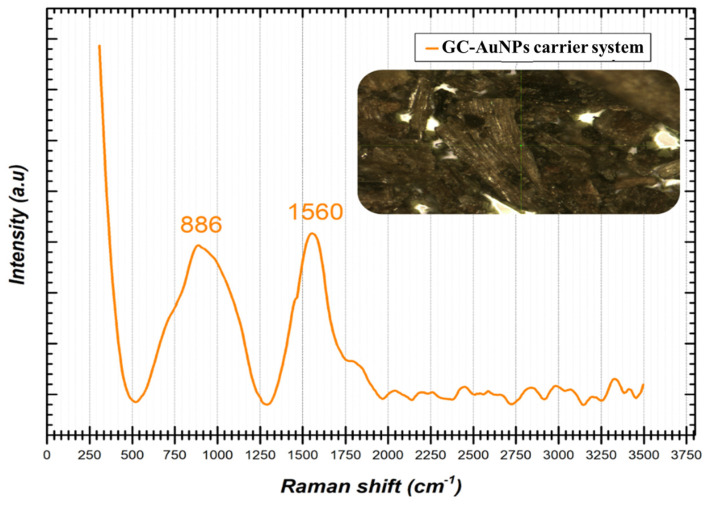
Raman spectrum of GC-AuNPs carrier system.

Figure 3 shows the characteristic Raman spectrum of the GC-AuNPs carrier system.

The Raman spectrum displayed in Figure 3 indicates the presence of two peak shifts identified at 886 cm^−1^ and 1560 cm^−1^, respectively. When gold nanoparticles are subjected to Raman analysis, certain specific shifts are expected to appear in the spectrum in the ranges of 200–400 cm^−1^ and 500–580 cm^−1^ [80]. In the present case, no spectral information could be identified in the previously mentioned intervals, but the presence in the spectrum of the two Raman shifts of significantly high intensity at 886 cm^−1^ and 1560 cm^−1^, respectively, can most probably be attributed to the vibrational modes specific to the interactions and the strong bonds between the surfaces of the AuNPs and the phytochemicals from the greater celandine sample [7]. It is important to note that the precise shifts in the Raman spectra may vary depending on the size, morphology, and surface chemistry of the AuNPs (single or hybrid), as well as on the experimental conditions. In addition, Raman spectroscopy is often used in combination with other complementary characterization techniques, so that a more detailed understanding of the properties of gold nanoparticles and how they interact with compounds in the plant extract of celandine can be obtained.

### 2.6. X-Ray-Diffraction Spectroscopy (XRD) 

X-Ray-Diffraction Spectroscopy (XRD) is a simple, fast, and non-destructive technique used to determine the phase composition and crystallographic data of materials [81].

**Figure 4:**
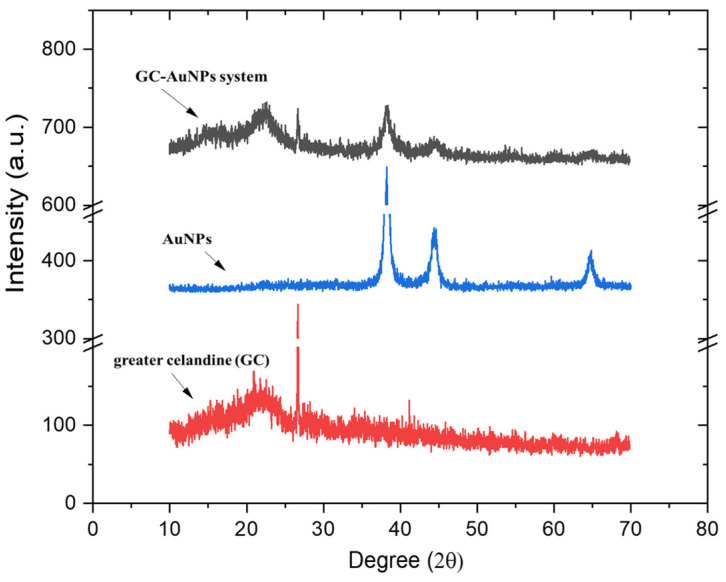
Powder XRD patterns of greater celandine, AuNPs, and CG-AuNPs carrier system.

Overlapped XRD patterns of the greater celandine, AuNPs, and GC-AuNPs carrier system are presented in Figure 4.

The XRD spectrum of AuNPs depicts defined peaks, evidencing a well-crystalline structure, with a crystallite mean size of 17 nm, as determined using the Scherrer equation [18].

The greater celandine pattern shows amorphous phases with poorly defined peaks in the (17–43°) range, associated with minerals and plant fibers. The XRD pattern of the GC-AuNPs carrier system exhibits, even if moderately weaker, peaks of herb components and AuNPs (Figure 4), thus confirming the formation of a new carrier system.

### 2.7. Scanning Electron Microscopy (SEM)

The comparative analysis of morpho-structural features was carried out using the SEM-EDX method.

**Figure 5:**
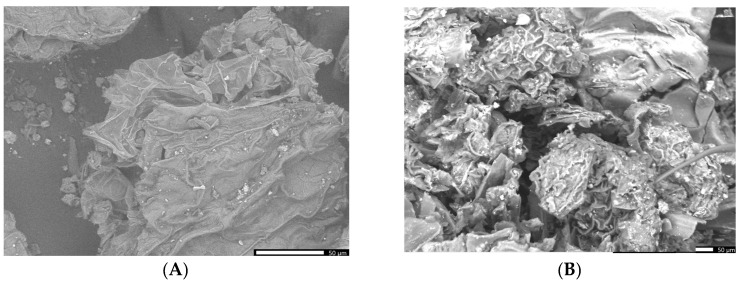

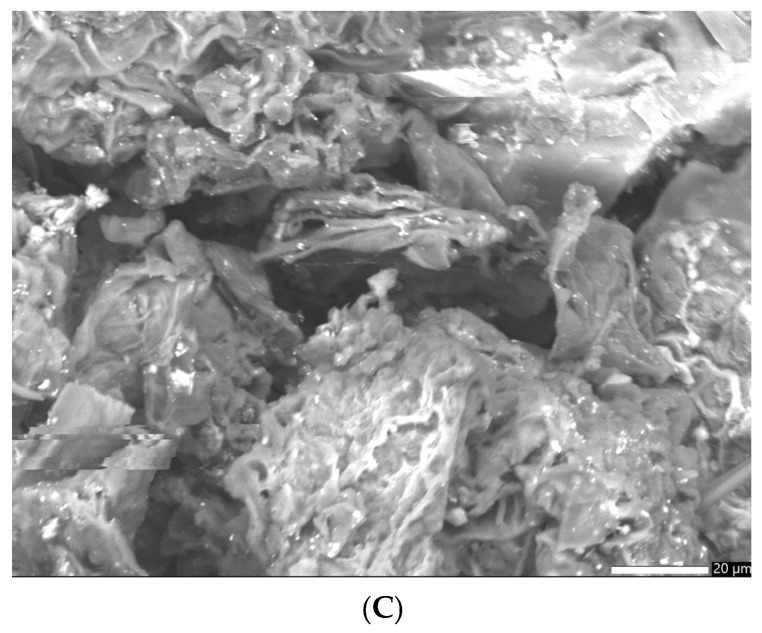
SEM images of the greater celandine (**A**) and CG-AuNPs carrier system (**B**,**C**).

It appears that the SEM image of the greater celandine (Figure 5A, low magnification (×850)) indicates the presence of a fibrous structure with large pores and irregular shape agglomerations of different-sized particles (average size: ~30 nm). The CG-AuNPs carrier system micrograph (Figure 5B,C low magnification (×850)) indicates that AuNPs and clusters of AuNPs (spherical shape, average size ~17 nm) were loaded in the herb particle pores.

**Figure 6:**
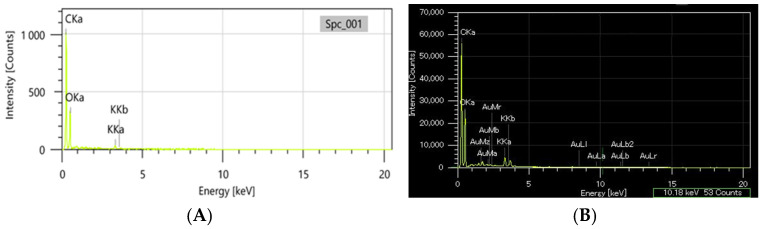
EDX composition of the greater celandine (**A**) and CG-AuNPs carrier system (**B**).

The EDX analysis was carried out comparatively on the greater celandine sample and the CG-AuNPs carrier system. The EDX spectra of the new carrier system are displayed beside the peaks corresponding to greater celandine (Figure 6A) and AuNPs (Figure 6B), indicating the achievement of the CG-AuNPs carrier system.

### 2.8. Dynamic Light Scattering (DLS)

Dynamic light scattering is a routine, accurate analytic method for the mean and distribution determination of nano- and micro-scale particles in dispersion. The hydrodynamic size, distribution, and stability of the GC-AuNPs carrier system were investigated via the DLS technique. The obtained results are shown in Table 4.

**Figure 7:**
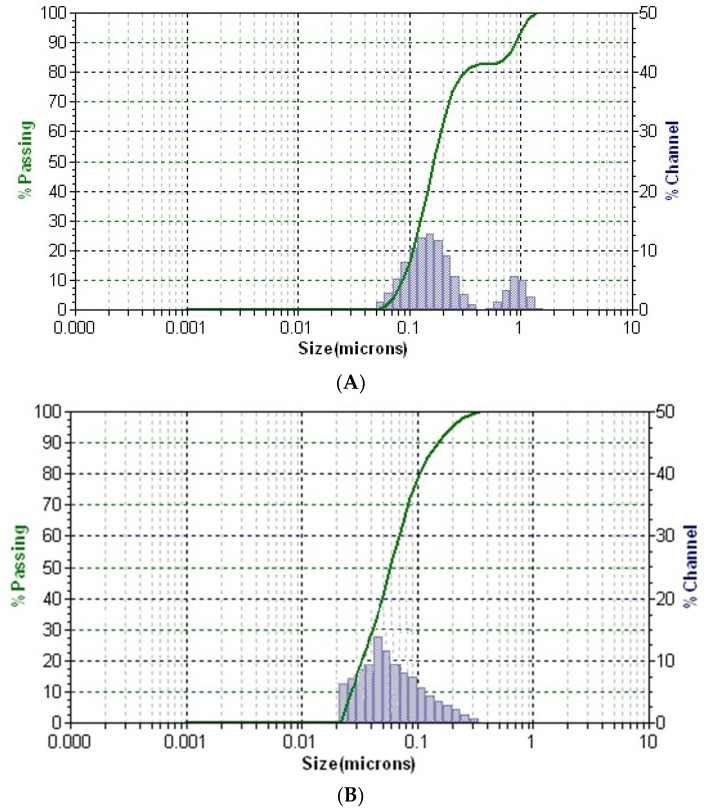

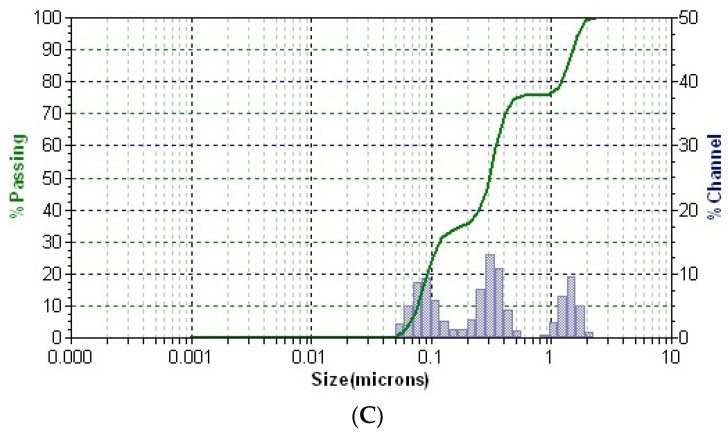
DLS patterns of the greater celandine (**A**), AuNPs (**B**), and CG-AuNPs carrier system (**C**).

The distribution of particles in solution for all samples is presented in Figure 7.

In the DLS pattern of the greater celandine sample (Figure 7A), there are two distinctive peaks corresponding to different hydrodynamic diameter values, which can be attributed to the fibrous structures and particles from the herb. According to the DLS analysis, the mean diameter of AuNPs is about 16 nm.

Conversely, the pattern of the CG-AuNPs carrier system (Figure 7C) exhibits three separate peaks, well-dispersed into a narrow range, indicating high stability. These peaks can be assigned to the presence of herb components (fibrous structures and particles) and AuNPs. It is worth noting that there was a significant shift in the sizes of AuNPs and herb components, which suggests that AuNPs were loaded into the herb pores. These findings agree with the results of the SEM study.

### 2.9. Thermal Properties

**Figure 8:**
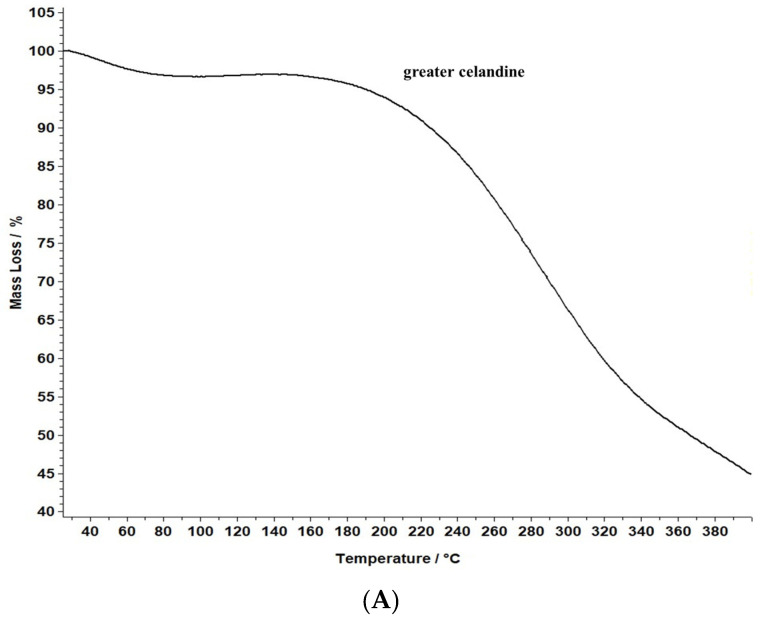

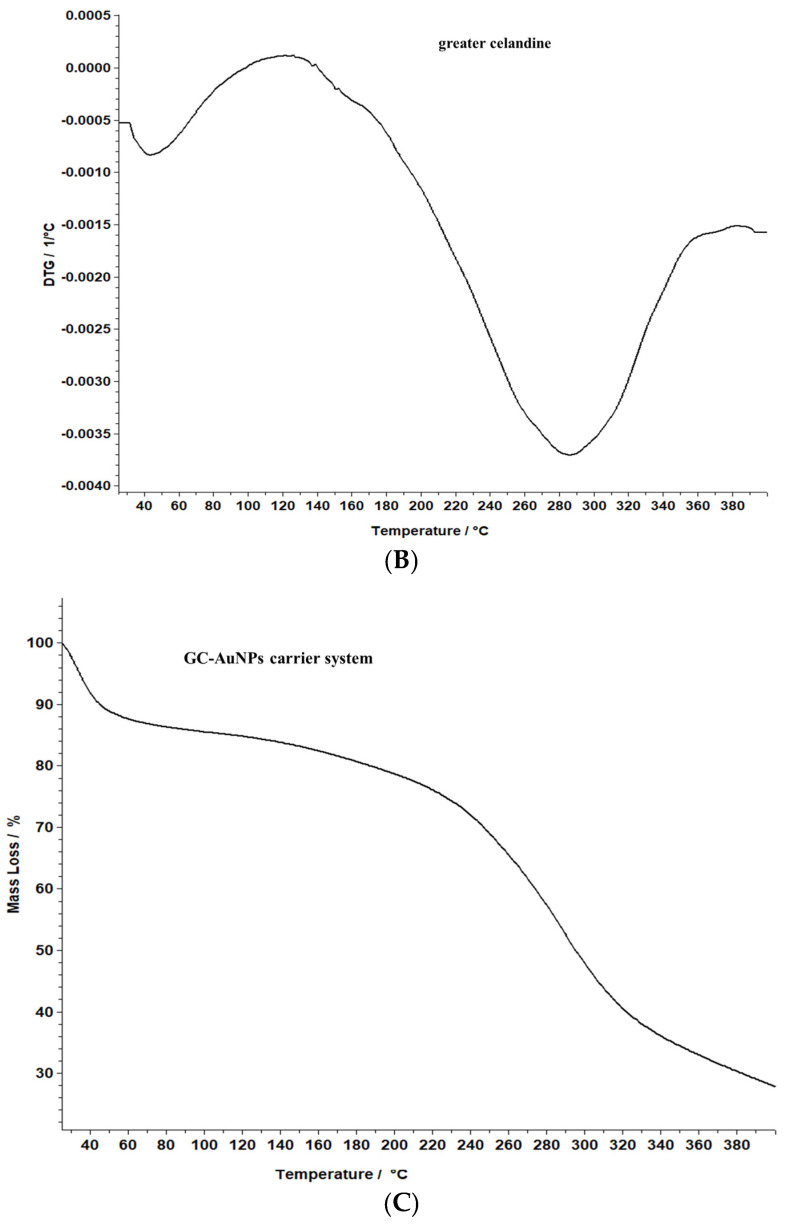

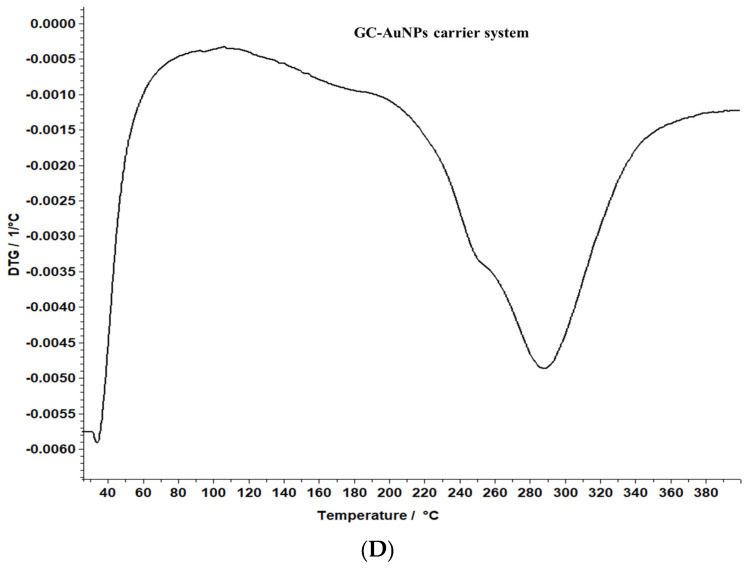
TG/DTG thermograms of the greater celandine sample (**A**,**B**) and CG-AuNPs carrier system (**C**,**D**).

A comparative evaluation was conducted to determine the thermal stability of the novel carrier system and greater celandine and to identify the chemical changes. The results are presented in Figure 8.

The data reveal that greater celandine demonstrated an endothermic process, resulting in a substantial weight loss (55%) in the temperature range of 150–180 °C due to moisture loss and decomposition of volatile compounds, carotenoids, alkaloids, and phenolics [82,83].

Similarly, the thermogravimetric curve of the novel carrier system indicated a noticeable weight loss (46%) at 190–220 °C, assigned to phytochemical decomposition. These changes in the differential thermogravimetric curve of the new carrier system may be linked to the loading of AuNPs in the herb particles, indicating a visible increase in the thermal stability of the CG-AuNPs carrier system. The findings provide valuable insight into the behavior of these samples, and could contribute to further research and development in this field.

### 2.10. In Vitro Dissolution Testing

In vitro dissolution assays are a ubiquitous technique in pharmacological development and quality control to predict the dissolution behavior and biological efficiency of drugs in the gastrointestinal tract [84,85]. However, due to the complex chemical composition, the bioavailability and performance assays for herbal formulations can be more challenging than for single compound products [84,85].

The pH value and time are key physiological factors with significant effects on the absorption of active biomolecules. Hence, a comparison study was performed between the dissolution profile of greater celandine and a newly prepared carrier system at pH values of 4 and 7 as a function of time.

**Figure 9:**
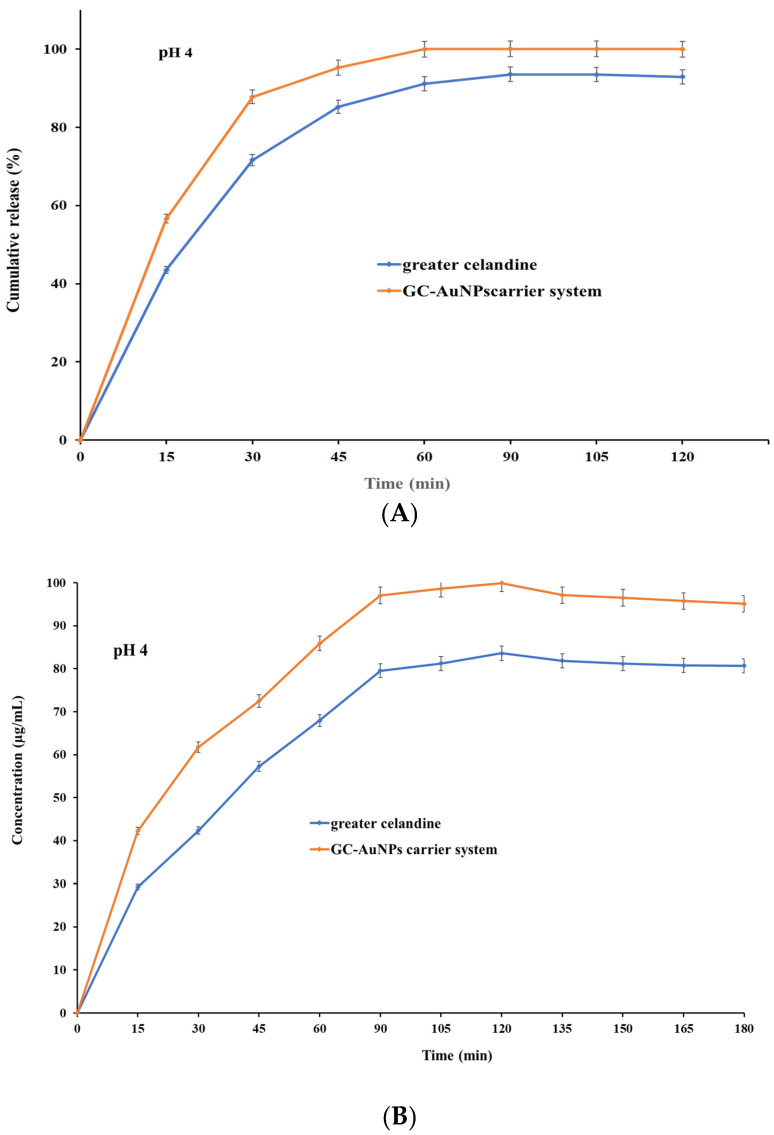

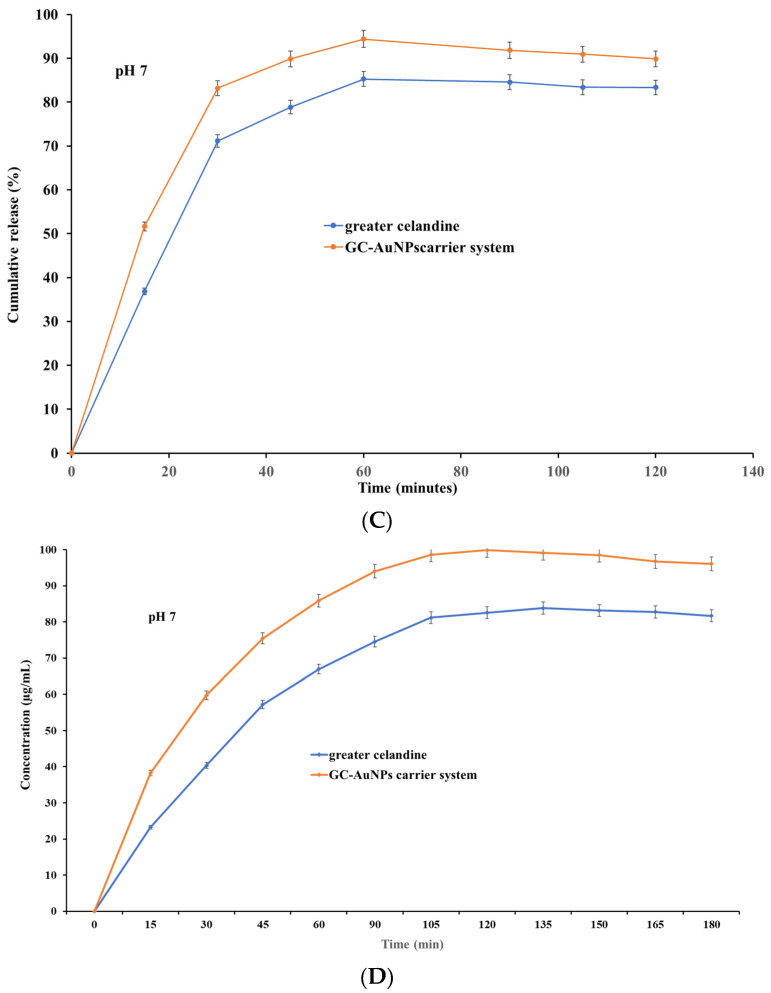
The dissolution profile of the greater celandine and CG-AuNPs carrier system.

The correlation between the pH value and dissolution rate is presented in Figure 9.

The results indicate that both samples exhibited, at pH 4, similar dissolution profiles and increased release (Figure 9 A) within 30 min (over 81% for GC and over 89% for carrier system), subsequently reaching a maximum value of 93.56% for the greater celandine (Figure 9B) and 99.99% for the CG-AuNPs carrier system at 60 min. Nevertheless, at pH 7, a notable difference appeared, specifically on the dissolution profile (Figure 9C). Furthermore, even though both samples still displayed a rapid release within 30 min, these values were significantly lower than at an acidic pH (about 71% for greater celandine and 83% for CG-AuNPs carrier system) (Figure 9D). The maximum release value was reached at 60 min for the greater celandine (85.26%) and the new carrier system (94.39%).

According to the results, the novel carrier system showed notably improved bioavailability compared to the greater celandine at both pH values investigated. This enhancement can be attributed to the specific surface modification under the employed experimental conditions. Additionally, a visible reduction in the dissolution rate (approximately 5% for the new carrier system and 8% for greater celandine) in a neutral environment (pH = 7) was observed. These unequivocal observations suggest that an acidic pH is more appropriate for the absorption of biomolecules. The findings of this study are highly significant and offer valuable insights that could significantly impact future studies on efficacy enhancement, as well as optimize the therapeutic outcome.

### 2.11. Screening of Antioxidant Activity

The evaluation of antioxidant potential for a specific herb formulation necessitates the selection of at least three appropriate antioxidant assays [86]. In vitro, non-competitive assays are widely acknowledged for their simplicity and accuracy in estimating the antioxidant potential of natural products [86,87].

**Figure 10:**
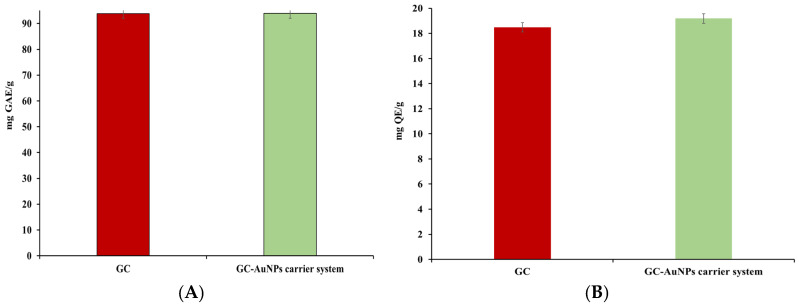

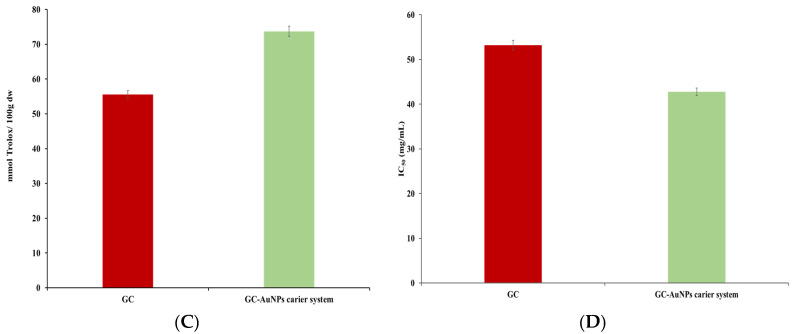
Graphic representation of total phenolic (**A**), flavonoid content (**B**), FRAP (**C**), and DPPH (**D**) results.

The antioxidant activity of a novel carrier system is attributed to a combination of collective bioactive phytochemicals from the greater celandine and the biological activity of the AuNPs. Consequently, four assays, namely, *total polyphenolic contents (TPCs)—Folin–Ciocalteu*, flavonoid content, FRAP, and DPPH were deemed adequate for estimating the antioxidant potential of the CG-AuNPs carrier system. The results are presented in Table 5 and Figure 10.

No significant differences were found in the TPCs or flavonoid assays for the novel carrier system compared to the greater celandine. However, in FRAP and DPPH tests, the CG-AuNPs carrier system exhibited higher antioxidant activity than the herb plant sample. Thus, the maximum value of the FRAP assay indicated an increase (up to 24%) for the carrier system. Similarly, the IC50 value was lower than that of the greater celandine by over 24%. These results can be attributed to modifications in the surface electric charge of metallic nanoparticles loaded into herb particles, as well as the synergistic action of AuNPs and the bioactive phytoconstituents [88,89].

This assay selection offers a comprehensive evaluation of the antioxidant potential of the carrier system, which is vital for the development of effective and safe antioxidant formulations [20,90].

## 3. Materials and Methods

All reagents were of analytical grade, purchased from commercial sources (Merck Millipore (Darmstadt, Germany), Sigma-Aldrich (München, Germany)), and used without further purification.

*Chelidonium majus* (greater celandine) samples (whole plant) were harvested in August 2023 from the area of Hunedoara County, Romania (geographic coordinates 45°43′04″N 22°53′13″E), and taxonomically authenticated at the University of Medicine and Pharmacy Craiova, Romania.

### 3.1. Plant Samples’ Preparation for Chemical Screening

The greater celandine samples underwent milling via a planetary Fritsch Pulverisette mill (Idar-Oberstein, Germany) (700 rpm for 10 min at 22 °C), followed by sieving through ASTM sieves. Only particles that passed through a 0.25 mm mesh sift were used in this study. Then, the plant samples were subjected to ultrasonic-assisted extraction (Elmasonic, Singen, Germany) under specific temperature, time, and ratio conditions (25 min, at 40 °C and 50 Hz, in methanol: chloroform = 1:1, *v*/*v*). The resulting extract was concentrated using a rotary evaporator, dissolved in MeOH (10 mL), centrifuged, and filtered. Subsequently, the extract samples were stored in a freezer until further use. All experiments were prepared in triplicate.

#### 3.1.1. GC-MS Analysis

Gas chromatography was carried out using a GCMS-QP2020NX Shimadzu apparatus with a ZB-5MS capillary column (30 m × 0.25 mm id × 0.25 µm) (Agilent Technologies, Santa Clara, CA, USA) and helium (flow rate of 1 mL/min.)

##### GC–MS Separation Conditions

The oven temperature was increased from 50 °C (kept for 2 min) to 300 °C (rate of 4 °C/min, hold for 5 min). The temperature of the injector was 290 °C, and the temperature at the interface was 217 °C. The mass of the compounds was registered at 70 eV of ionization energy. The mass spectrometer was source-heated at 225 °C, and the MS Quad was heated at 155 °C. Phytochemicals were identified based on their mass spectra compared to the NIST0.2 mass spectra library database (USA National Institute of Science and Technology software, (NIST, Gaithersburg, MD, USA) and the literature review.

#### 3.1.2. Mass Spectrometry

The MS experiments were performed using an EIS-QTOF-MS (Bruker Daltonics, Bremen, Germany). The mass spectra were acquired in positive ion mode in a mass range of 50–3000 *m*/*z*. The scan speed was 2.1 scans/s, the collision energy was 10 ÷ 85 eV, and the temperature of the source block was 80 °C. Compounds were identified based on their mass spectra, then compared to the NIST 3.0 database mass spectra library database (USA National Institute of Science and Technology software) (NIST, Gaithersburg, MD, USA) and the literature review.

### 3.2. CG-AuNPs Carrier System Preparation

#### 3.2.1. The synthesis of AuNPs was achieved according to the *procedure* described in *our earlier* publication [18]

#### 3.2.2. CG-AuNPs Carrier System

The greater celandine sample (whole plant dried) and AuNPs solution were mixed (1:2.5 mass ratio) under continuous stirring for 6 h at room temperature (22 °C). The emerging suspension was centrifuged, filtered, and then dried at 40 °C for 6 h. Each experiment was repeated three times.

### 3.3. Characterization of Novel Carrier System and Raw Materials

#### 3.3.1. Fourier Transform Infrared (FT-IR) Spectroscopy

FT-IR spectra of the CG-AuNPs carrier system and its components in the solid phase were recorded on a Fourier transform infrared spectrometer (Shimadzu AIM-9000 with ATR devices).

#### 3.3.2. XRD Spectroscopy

Data on the phase composition were obtained on a Bruker AXS D8-Advance X-ray diffractometer (Bruker AXS GmbH, Karlsruhe, Germany) equipped with a rotating sample stage; an Anton Paar TTK low-temperature cell (−180 °C ÷ 450 °C); a high-vacuum, inert atmosphere; and relative humidity control. The average crystallite size and phase content were determined using the whole-pattern profile-fitting method (WPPF).

#### 3.3.3. Scanning Electron Microscopy (SEM)

Morpho-structural investigations were carried out using an SEM-EDS system (JEOL JSM-IT200 Field Emission, Nieuw-Vennep, The Netherlands) equipped with a high-resolution electron gun and an energy-dispersive X-ray spectrometer (EDS).

#### 3.3.4. Dynamic Light Scattering (DLS) Particle Size Distribution Analysis

The DLS analysis was conducted with a scattering angle of 172 °C at room temperature (22 °C) using a Microtrac/Nanotrac 252 (Montgomeryville, PA, USA) instrument. Each experiment was repeated three times.

#### 3.3.5. Thermal Analysis

The thermal stability study of the novel carrier system and herb sample was performed in a dynamic air atmosphere (20 mL/min, synthetic air) at a temperature range of 25 ÷ 400 °C and a heating rate of 10 ° C/min using a Thermal Analyzer produced by METTLER TOLEDO, model TGA/DSC3^+^ STARe System. The DSC analysis was performed in an air atmosphere (50 mL/min), at a temperature range of 25–400 °C, on a DSC 3+ Mettler Toledo.

### 3.4. Antioxidant Activity

In vitro antioxidant potential screening of the novel carrier system and herb sample were examined using four distinct tests: Folin–Ciocalteu assay; flavonoid content assay; 2,2-diphenyl-1-picrylhydrazyl; (DPPH) radical scavenging assay; and ferric reducing antioxidant power assay (Frap).

#### 3.4.1. Folin–Ciocalteu Assay

The antioxidant activity of *Chelidonium majus* and GC-AuNPs was determined using UV-VIS spectrophotometry (DLAB SP-UV1000, Penjuru, Singapore), according to the experimental procedure described in the literature [91]. The results are expressed in gallic acid equivalents (mg GAE/g sample). Sample concentrations were calculated based on the linear equation obtained from the standard curve (y = 0.0015x + 0.2134) and the correlation coefficient (R^2^ = 0.9971).

#### 3.4.2. The Flavonoid Content Assay

The flavonoid contents from both samples were determined according to the experimental procedure adapted from the literature [92].

The absorbance was measured at 510 nm using a UV-Vis spectrometer (DLAB SP-UV1000). The flavonoid content was expressed in quercetin equivalents (mg QE/g) using a quercetin standard calibration curve between 12.5 mg/mL and 100 mg/mL in methanol. Sample concentrations were calculated based on the linear equation obtained from the standard curve (y = 0.0083x + 0.1114) and the correlation coefficient (R^2^ = 0.9961).

#### 3.4.3. Ferric Reducing Antioxidant Power Assay (FRAP)

The ferric reducing/antioxidant activity (FRAP) of the sample was determined spectrophotometrically using a Ferric Reducing Antioxidant Power (FRAP) Assay Kit (MAK369-1KT, Sigma-Aldrich). The absorbance was measured at 594 nm using a UV-Vis spectrometer (Elabscience^®^, Houston, TX, USA). The results were expressed in Trolox equivalents (mmol Trolox equivalents/100 g dry weight (dw)).

#### 3.4.4. DPPH Radical Scavenging Assay

The radical scavenging properties of the novel carrier system and herb sample were assessed according to the procedure described in *our earlier* publication [93]. The results were used to calculate and obtain the IC_50_ (mg/mL).

All experiments for antioxidant activity screening were performed in triplicate.

### 3.5. In Vitro Dissolution Test

The dissolution profiles of the greater celandine (0.5 g ± 0.012) and CG-AuNps carrier system (0.5 g ± 0.016) were determined using a 708-DS Dissolution-Agilent Technologies (Santa Clara, CA, USA). The tests were conducted under strict conditions: a temperature of 37 ± 0.25 °C, a speed of 100 rpm, and two buffers of pH 4 and pH 7, respectively, to simulate the gastric and intestinal fluids [94].

Sink conditions were rigorously maintained throughout the test.

During the experiment, samples of dissolution medium (5 mL) were collected at different times (15–120 min). The cumulative drug released against time was determined spectrophotometrically (UV-Vis Perkin-Elmer Lambda 35 (Perkin Elmer, Waltham, MA, USA).

Triplicate samples were analyzed at each time point. The mean values of the samples and the standard deviation were calculated [95].

#### Preparation of the Curves of the Concentrations for the Compound Dissolution Profile

Different solution concentrations (in the range of 0.00 and 0.30 mg/mL) were prepared from each sample (greater celandine and CG-AuNP carrier system, respectively). Then, calibration curves were plotted. The amount of compound released was obtained from the standard curve of the concentration versus its absorbency. The correlation coefficients at pH = 4 were: R2 = 0.9978 (greater celandine) and R2 = 0.9986 (CG-Au NPs carrier system), and at pH = 7, R2 = 0.9991 (greater celandine) and R2 = 0.99984 (CG-Au NPs carrier system). This demonstrates the good linear relationship of the data.

The compound release was calculated according to Equation (1) [96]:


(1) $$CDR\;\left(\%\right)=\;\frac{amount\;of\;released\;compund\;at\;time\;n\;(g)}{amount\;compound\;used\;as\;raw\;materials\;(g)}\;\times \;100$$

### 3.6. Statistical Analysis

Statistical analysis was conducted using IBM SPSS Statistics 21.0 for Windows (SPSS Inc.). Each experimental set was performed in triplicate, using one-way analysis of variance (ANOVA) without replication with Scheffe’s post hoc test for comparison; *p* < 0.05 was taken as statistically significant. Data are presented as the means ± SD.

## 4. Conclusions

This study presents the low-molecular-mass-metabolite profiling of *Chelidonium majus* growing wild in Romania, followed by the development and in vitro evaluation of the antioxidant and release of a novel carrier system prepared using this medicinal plant and AuNPs. Various analytical methods, including FTIR, Raman, XRD, DLS, and SEM-EDX, were employed to confirm the preparation of the carrier system. The TG/DTG study results demonstrated that the GC-AuNPs carrier system had superior thermal behavior compared to the herb sample. The study indicates that this novel carrier system had significantly enhanced antioxidant activity and an improved release rate. These results suggest its auspicious potential as a promising candidate for various applications.

## Figures and Tables

**Table 1:** Main biomolecules identified by GC–MS analysis of *Chelidonium majus* sample.

No	Compound Name	Retention Time (RT)	Area%	Ref.
1	5-hydroxymethylfurfural	6.72	0.52	[39]
2	limonene	10.16	0.32	[40]
3	neoxanthin	15.42	18.44	[41]
4	anethole	18.91	11.13	[40]
5	sanguinarine	20.02	0.49	[42]
6	beta-carotene	21.06	0.51	[41]
7	dihydroberbine	24.53	0.36	[30]
8	chelidonine	25.12	23.48	[30]
9	dihydrosanguinarine	26.77	0.58	[30]
10	dihydrochelerythrine	29.01	2.43	[30]
11	chelerythrine	30.78	1.12	[42]
12	oxysanguinarine	31.67	27.35	[42]
13	angoline	35.89	0.47	[36]

**Table 2:** Biomolecules identified in *Chelidonium majus* sample through MS analysis.

No	Tentative of Identification	Formula	Category	*m*/*z* Detected	Theoretic *m*/*z*	Ref.
1	acetic acid	C_2_H_4_O_2_	organic acid	61.07	60.05	[43]
2	glycine	C_2_H_5_NO_2_	amino acids	76.05	75.07	[31]
3	alanine	C_3_H_7_NO_2_	amino acids	90.10	89.09	[31]
4	dihydroxyacetone	C_3_H_6_O_3_	ketones	91.09	90.08	[43]
5	choline	C_5_H_14_NO^+^	cholines	105.16	104.17	[11]
6	serine	C_3_H_7_NO_3_	amino acids	106.09	105.09	[31]
7	histamine	C_5_H_9_N_3_	amines	112.14	111.15	[11]
8	proline	C_5_H_9_NO_2_	amino acids	116.14	115.13	[31]
9	valine	C_5_H_11_NO_2_	amino acids	118.16	117.15	[31]
10	succinic acid	C_4_H_6_O_4_	organic acids	119.08	118.09	[15]
11	threonine	C_4_H_9_NO_3_	amino acids	120.13	119.12	[31]
12	nicotinic acid	C_6_H_5_NO_2_	organic acids	124.11	123.11	[15]
13	5-hydroxymethylfurfural	C_6_H_6_O_3_	furans	127.12	126.11	[42]
14	isoleucine	C_6_H_13_NO_2_	amino acids	132.18	131.17	[31]
15	asparagine	C_4_H_8_N_2_O_3_	amino acids	133.13	132.12	[31]
16	aspartic acid	C_4_H_7_NO_4_	amino acids	134.11	133.10	[31]
17	malic acid	C_4_H_6_O_5_	organic acids	135.08	134.09	[33]
18	limonene	C_10_H_16_	terpenoids	137.23	136.23	[40]
19	tyramine	C_8_H_11_NO	amines	138.19	137.18	[11]
20	salicylic acid	C_7_H_6_O_3_	organic acids	139.11	138.12	[33]
21	glutamic acid	C_5_H_9_NO_4_	amino acids	148.14	147.13	[31]
22	anethole	C_10_H_12_O	anisoles	149.21	148.20	[40]
23	vanillin	C_8_H_8_O_3_	aldehydes	153.14	152.15	[33]
24	gentisic acid	C_7_H_6_O_4_	phenolic acids	155.11	154.12	[33]
25	*p*-coumaric acid	C_9_H_8_O_3_	phenolic acids	165.15	164.16	[33,38]
26	vanillic acid	C_8_H_8_O_4_	phenolic acids	169.16	168.15	[33]
27	gallic acid	C_7_H_6_O_5_	phenolic acids	171.13	170.12	[11,15]
28	trans-aconitic acid	C_6_H_6_O_6_	organic acids	175.12	174.11	[33]
29	caffeic acid	C_9_H_8_O_4_	phenolic acids	181.15	180.16	[15]
30	tyrosine	C_9_H_11_NO_3_	amino acids	182.19	181.19	[31]
31	chelidonic acid	C_7_H_4_O_6_	organic acids	185.11	184.10	[15]
32	quinic acid	C_7_H_12_O_6_	phenolic acids	193.18	192.17	[15,33]
33	ferulic acid	C_10_H_10_O_4_	phenolic acids	195.19	194.18	[15,39]
34	aporphine	C_17_H_17_N	alkaloids	236.33	235.32	[33]
35	linoleic acid	C_18_H_32_O_2_	fatty acids	281.41	280.40	[11]
36	oleic acid	C_18_H_34_O_2_	fatty acids	283.51	282.50	[11]
37	luteolin	C_15_H_10_O_6_	flavonoids	297.23	286.24	[11]
38	sparteine	C_15_H_26_N_2_	alkaloids	235.39	234.38	[11]
39	palmitic acid	C_16_H_32_O_2_	fatty acids	257.43	256.42	[43]
40	9-octadecenoic acid	C_18_H_34_O_2_	fatty acids	283.49	282.50	[43]
41	quercetin	C_15_H_10_O_7_	flavonoids	303.23	302.23	[11]
42	isorhamnetin	C_16_H_12_O_7_	flavonoids	317.25	316.26	[33]
43	coptisine	C_19_H_14_NO_4_^+^	alkaloids	321.29	320.30	[6]
44	stylopine	C_19_H_17_NO_4_	alkaloids	324.31	323.30	[7,11]
45	scoulerine	C_19_H_21_NO_4_	alkaloids	328.41	327.40	[11]
46	sanguinarine	C_20_H_14_NO_4_^+^	alkaloids	333.31	332.30	[6,11]
47	dihydrosanguinarine	C_20_H_15_NO_4_	alkaloids	334.29	333.30	[11]
48	berberine	C_20_H_18_NO_4_^+^	alkaloids	337.41	336.40	[6,11]
49	canadine	C_20_H_21_NO_4_	alkaloids	340.39	339.40	[6,11]
50	corydine	C_20_H_23_NO_4_	alkaloids	342.41	341.40	[11]
51	magnoflorine	C_20_H_24_NO_4_^+^	alkaloids	343.41	342.40	[11]
52	oxysanguinarine	C_20_H_13_NO_5_	alkaloids	348.29	347.30	[11]
53	chelerythrine	C_21_H_18_NO_4_^+^	alkaloids	349.39	348.40	[11,33]
54	dihydrochelerythrine	C_21_H_19_NO_4_	alkaloids	350.41	349.40	[11]
55	chelidonine	C_20_H_19_NO_5_	alkaloids	354.39	353.40	[6,11]
56	rosmarinic acid	C_18_H_16_O_8_	phenolic acids	361.29	360.30	[33]
57	dihydrochelirubine	C_21_H_17_NO_5_	alkaloids	364.41	363.40	[11]
58	allocryptopine	C_21_H_23_NO_5_	alkaloids	370.39	369.40	[6,11]
59	angoline	C_22_H_21_NO_5_	alkaloids	380.41	379.40	[11]
60	1-hexacosanol	C_26_H_54_O	alcohols	383.69	382.70	[15]
61	sanguilutine	C_23_H_24_NO_5_^+^	alkaloids	395.39	394.40	[11]
62	dihydroberbine	C_20_H_19_NO_4_	alkaloids	338.41	337.40	[15]
63	chlorogenic acid	C_16_H_18_O_9_	phenolic acids	355.32	354.31	[11]
64	quercetol C	C_22_H_24_O_5_	flavonoids	369.39	368.40	[38]
65	ergosterol	C_28_H_44_O	sterols	397.61	396.60	[15]
66	stigmasterol	C_29_H_48_O	sterols	413.71	412.70	[38]
67	β sitosterol	C_29_H_50_O	sterols	415.69	414.70	[38]
68	nonacosanol	C_29_H_60_O	alcohols	425.81	424.80	[15]
69	hyperoside	C_21_H_20_O_12_	flavonoids	465.39	464.40	[11]
70	beta-carotene	C_40_H_56_	carotenoids	537.91	536.90	[11]
71	zeaxanthin	C_40_H_56_O_2_	carotenoids	569.89	568.90	[11]
72	neoxanthin	C_40_H_56_O_4_	carotenoids	601.91	600.90	[11]
73	chlorophyll a	C_55_H_72_MgN_4_O_5_	carotenoids	894.49	893.50	[11]
74	chlorophyll b	C_55_H_70_MgN_4_O_6_	carotenoids	908.51	907.50	[11]

**Table 3:** The characteristic vibrational bands attributed to various biomolecule categories from the *Chelidonium majus* sample.

Biomolecule Category	Characteristic Vibrational Bands (cm ^−1^)	Ref.
alkaloids	3362; 1598; 1646; 1402; 1375; 741; 663	[69]
amino acids	3380; 2358, 2128; 1751; 1675; 1665; 1649; 1632	[70]
phenolic acids	1662; 1727; 1640; 1521; 1410; 1363; 1262; 1168; 1091; 947	[18,71]
flavonoids	3234; 3082; 1655; 1618; 1583; 1465; 1415; 1372; 1274; 1079; 771; 536	[72]
fatty acids	3601; 3018; 2959; 2922; 2874; 1703; 1352; 1247; 723	[73]
carotenoids	2922; 1632; 1385; 965	[74]
phytosterols	3427; 2940; 2838; 1752; 1467; 1382; 1188; 1065; 990; 883; 742	[75,76]

**Table 4:** The DLS mean hydrodynamic diameter values of the GC-AuNPs carrier system and components.

Sample	Diameters (µm)	Width (µm)
greater celandine	0.9610	0.3190
	0.2555	0.1089
AuNPs	0.01675	0.0641
GC-AuNPs carrier system	1.4530	0.5040
	0.3250	0.1413
	0.0892	0.0579

**Table 5:** The result of the selected antioxidant assay for the greater celandine and CG-AuNPs carrier system.

Sample Name	Total Phenolic Content (mg/g GAE)	Flavonoid Content (mg QE/g)	FRAP (mmol Trolox/100g dw)	IC_50_ (mg/mL)
GC	93.87 ± 0.028	18.48 ± 0.034	55.57 ± 0.011	53.23 ± 0.012
GC-AuNPs system	93.32 ± 0.033	19.18 ± 0.062	73.74 ± 0.014	42.78 ± 0.036

## Data Availability

Data are contained within the article.

## Supplementary Materials

The following supporting information can be downloaded at: https://www.mdpi.com/article/10.3390/plants13050734/s1, Figure S1: TIC chromatogram of *Chelidonium majus* sample.; Figure S2: The mass spectra of *Chelidonium majus* sample.
