# Supplementary material for: Romanian Wild-Growing Chelidonium majus—An Emerging Approach to a Potential Antimicrobial Engineering Carrier System Based on AuNPs: In Vitro Investigation and Evaluation
Source: Plants (Basel). 2024 Mar 5;13(5):734. doi: 10.3390/plants13050734 (PMC10934343; doi:10.3390/plants13050734)
Supplement: Supplementary file 1 [file plants-13-00734-s001.zip › plants-2854757-supplementary.pdf]

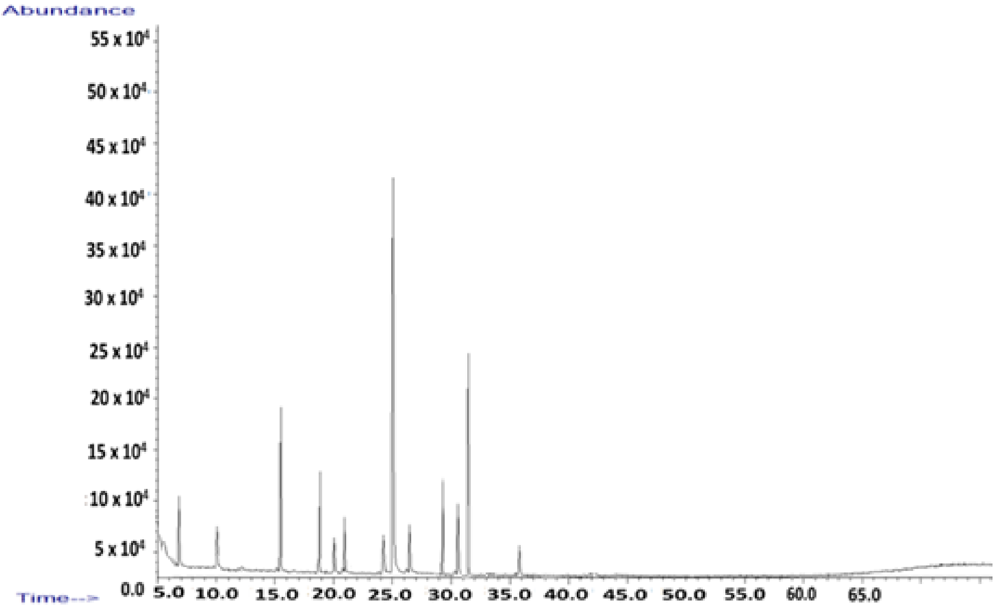

Figure S1. TIC chromatogram of *Chelidonium majus* sample.

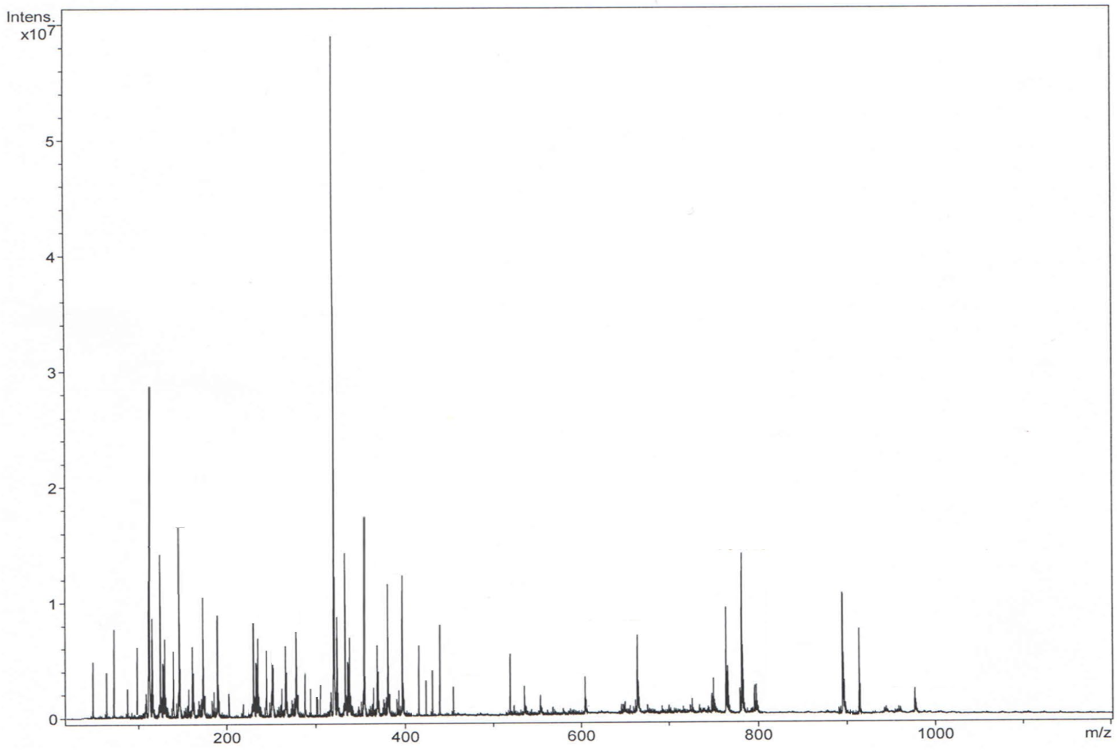

Figure S2. The mass spectra of *Chelidonium majus* sample.
